# Novel concept for posterior crossbite correction: Preliminary results

**DOI:** 10.1007/s00056-023-00468-0

**Published:** 2023-05-04

**Authors:** Dirk Wiechmann

**Affiliations:** 1 Department of Orthodontics, Hannover Medical School (MHH), Hannover, Germany; 2 Private Practice, Lindenstr. 44, 49152 Bad Essen, Germany

**Keywords:** Fixed orthodontic appliances, Lingual orthodontics, Expansion/compression archwires, Mandibular compression, Palatal expansion technique, Festsitzende kieferorthopädische Apparaturen, Linguale Kieferorthopädie, Expansions‑/Kompressionsbögen, Kompression des Unterkiefers, Palatinale Erweiterungsmethode

## Abstract

**Purpose:**

The efficiency of dentoalveolar compensation involving both jaws for posterior crossbite correction using computer-aided design/computer-aided manufacturing (CAD/CAM) expansion and compression archwires was evaluated. Treatment outcome was tested against the null hypothesis that the transverse correction achieved would be significantly smaller than planned.

**Methods:**

This retrospective study included 64 patients (mean age 23.5 years, median 17.0, minimum/maximum: 9.0/63.0, standard deviation 13.7) with uni- or bilateral posterior crossbite. In all consecutively debonded patients, expansion and/or compression archwires were used for dentoalveolar correction involving both jaws. Plaster casts prior to (T1) and following treatment (T2) with completely customized lingual appliances (CCLA) were compared with the treatment plan represented by an individual target set-up. The statistical analysis was carried out using the Schuirmann TOST (two one-sided t‑tests) equivalence test on the basis of a one-sample t‑test with α = 0.025 to one side. The non-inferiority margin was set at δ = 0.5 mm.

**Results:**

All posterior crossbites could be corrected by dentoalveolar compensation involving both jaws. The mean total correction achieved was 6.9 mm (mean maxillary expansion: 4.3 mm/mean mandibular compression: 2.6 mm) with a maximum of 12.8 mm. The transverse corrections achieved in both arches at T2 were equivalent to the planned corrections in the set-up (*p* < 0.001).

**Conclusion:**

The results of this study indicate that CAD/CAM expansion and compression archwires can be an efficient tool to achieve the desired correction in patients with a posterior crossbite even in more severe cases.

## Abstract (de)

**Zielsetzung:**

Untersucht wurde die Effizienz einer dentoalveolären Kompensation aus beiden Kiefern bei der Korrektur eines seitlichen Kreuzbisses mithilfe von CAD/CAM(„computer-aided design/computer-aided manufacturing“)-gefertigten Expansions- und Kompressionsbögen. Das Behandlungsergebnis wurde gegen die Nullhypothese getestet, dass die erzielte transversale Korrektur signifikant kleiner ist als die geplante.

**Methoden:**

In dieser retrospektiven Studie wurden 64 Patienten (Durchschnittsalter: 23,5 Jahre, Median: 17,0, Minimum/Maximum: 9,0/63,0, SD [„standard deviation“]: 13,7) mit ein- oder beidseitigem seitlichen Kreuzbiss nachuntersucht. Nach konsekutivem Debonding wurden allen Patienten Expansions- und/oder Kompressionsbögen zur dentoalveolären Korrektur aus beiden Kiefern eingesetzt. Die Gipsmodelle vor (T1) und nach der Behandlung (T2) mit einer vollständig individuellen lingualen Apparatur (VILA) wurden mit dem Behandlungsplan, repräsentiert durch ein individuelles Ziel-Set-up, verglichen. Zur statistischen Auswertung wurde der TOST(„two one-sided t‑tests“)-Äquivalenztest nach Schuirmann auf der Basis eines einseitigen t‑Tests mit α= 0,025 durchgeführt. Die Nichtunterlegenheitsgrenze wurde mit δ = 0,5 mm definiert.

**Ergebnisse:**

Alle seitlichen Kreuzbisse konnten durch dentoalveoläre Kompensation aus beiden Kiefern überstellt werden. Die durchschnittlich erzielte transversale Korrektur betrug 6,9 mm (durchschnittliche Oberkieferexpansion: 4,3 mm/durchschnittliche Unterkieferexpansion: 2,6 mm) mit einer Maximalkorrektur von 12,8 mm. Die in beiden Kiefern erzielte transversale Korrektur (T2) war äquivalent zu der im Set-up geplanten (*p* < 0,001).

**Fazit:**

Die Ergebnisse dieser Studie zeigen, dass mithilfe von CAD/CAM-Expansions- und Kompressionsbögen eine effiziente Korrektur eines seitlichen Kreuzbisses auch in schwierigeren Fällen erfolgen kann.

Uni- or bilateral posterior crossbite is a frequent finding in orthodontic patients, with a global prevalence of up to 30% in the mixed and permanent dentition [2, 17]. Etiological factors that have been described include sucking habits [25, 27, 29, 33], upper airway obstruction [34], incorrect orofacial functions [35] or short frenulum linguae [32], all of which favour a low tongue position [26, 28, 46]. In most cases of uni- or bilateral posterior crossbite, not only the maxillary arch appears to be too narrow, but also the mandibular arch is overexpanded [4, 14, 20, 22, 23, 28, 31, 36, 41]. In 2002, Thilander and Lennartson concluded in their paper on the stability of posterior crossbite correction: “It should be borne in mind that narrow upper and broad lower dimensions will result in failure if an expansion appliance is used in the upper jaw only” [41]. Five years later, Bartzela and Jonas found the same result when looking at long-term stability after crossbite correction and concluded: “In patients where a broad lower arch is a cofactor in the etiology of the lateral crossbite, the treatment approach should be focused on both arches and not be limited to the constricted upper arch” [4].

Nonetheless, posterior crossbites are corrected today mainly by expanding the maxillary arch, using different techniques without or with surgical assistance (Schwarz plate, rapid maxillary expansion (RME), mini-screw assisted rapid palatal expansion (MARPE), surgically assisted rapid palatal expansion (SARPE), or one- or two-piece surgical maxillary segmentation). In most cases the laterally overexpanded mandibular arch is not corrected or even further expanded using conventional fixed labial appliances [39]. Only a few case reports demonstrate the possibility of compressing the mandibular lateral region with labial appliances, by either mandibular extractions or relatively thick auxiliary lingual arches used to achieve the attempted correction [22, 31].

Lingual appliances offer biomechanical advantages for effective modification of the upper and lower archform, as their archwires are substantially shorter (20–25%) than labial ones in the area from second molar to second molar. Therefore, assuming the same deflection, for example in the area of the first molars, the corrective force delivered by a lingual appliance is higher. Completely customized lingual appliances (CCLAs) use stainless steel archwires precision-bent by a bending robot [47]. Expansion and compression archwires are designed and manufactured with the help of computer-aided design/computer-aided manufacturing (CAD/CAM) technologies. Extra-torque as well as expansion and compression bends are incorporated as part of the wire manufacture in the anterior segment from canine to canine [5]. Therefore, the two separate bending algorithms have to be combined and adapted to the modified e‑modulus of an archwire already plastically deformed by a first bend. When defining the perfect wire shape in the computer (computer-aided design), the orthodontist can modify the posterior transverse dimension of these archwires by expanding or compressing them. For posterior crossbite correction, an expansion in the maxilla of 1, 2 or 3 cm in the area of the first molar is possible depending on the individual biological situation. Accordingly, in the mandible a 1 or 2 cm compression can be designed (Fig. 1). These archwires have been used in one orthodontic specialist practice in Germany for several years for patients of all age groups treated with CCLAs. For patients with a posterior crossbite, the individual treatment plan expressed in an individual set-up followed the recommendations of Thilander and Lennartsson [41]/Bartzela and Jonas [4] to correct posterior crossbite from both arches.

**Fig. 1:**
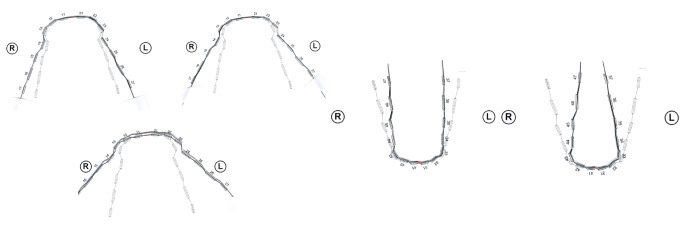
Completely customized lingual appliance (CCLA) archwires with 1, 2 or 3 cm expansion in the maxilla (*left*) and 1 or 2 cm of compression in the mandible (*right*) in the area of the first molar. The corrective bends are incorporated in the anterior segment between 3 and 3 VILA(vollständig individuelle linguale Apparatur)-Bögen mit 1, 2 bzw. 3 cm Expansion im Oberkiefer (*links*) und 1 bzw. 2 cm Kompression im Unterkiefer, gemessen am jeweils ersten Molaren. Die Korrekturbiegungen wurden im anterioren Bereich von 3–3 eingebogen

In order to evaluate the effectiveness of these CAD/CAM expansion and compression archwires, the outcome was tested against the null hypothesis that the transverse correction achieved would be significantly smaller than planned on the individual set-up.

## Subjects and method

This retrospective cohort study was approved by the ethical committee of the Hannover Medical School, Hannover, Germany (3151-2016). All patients were treated in one orthodontic specialist practice and consecutively debonded in the period from 2019–2021. Of the 1046 patients who received lingual treatment in this period with a CCLA (WIN, DW Lingual Systems GmbH, Bad Essen, Germany), 67 patients exhibited a posterior crossbite of at least four antagonistic teeth pretreatment. Three patients were excluded from the sample, as the posterior crossbite was partly corrected by class III surgery. No patient from this consecutive sample was excluded for any other reason. The average age of the 64 patients evaluated in this study at T1 was 23.5 years (median 17.0 years, minimum 9.0/maximum 63.0; standard deviation [SD] 13.7).

The individual treatment plan expressed in the set-up, consisted in maxillary expansion and mandibular compression in the area of the crossbite in order to harmonize the shape of both arches. This concept was also applied for the 23 patients which had a two-phase treatment with a fixed Hyrax expander, a removable Schwarz plate or a removable functional appliance, which were used before lingual treatment. To achieve these treatment objectives, 0.016″ × 0.024″ stainless steel expansion and compression archwires were used based on the clinical assessment by the treating orthodontist. No cross-elastics were used for crossbite correction. The transverse dimension was measured in the area of the biggest transverse discrepancy, which normally was at the second premolar or the first or second molar. The measurements were taken with a digital calliper on plaster casts before (T1) and after treatment (T2) as well as on the individual set-up cast.

## Statistical analysis

To assess the patient characteristics as well as the amount of the transverse correction, the measurement data were analysed descriptively, using mean and standard deviation (SD), as well as median, minimum and maximum values (min–max) at the various time points under consideration (T1 and T2). The primary endpoints were the transverse correction in maxillary and mandibular arch as well as the total transverse correction.

To analyse if the transverse correction at the end of treatment was not significantly less than planned on the set-up, a test for equivalence, based on the difference (setup–T2), was used to assess whether the mean difference and corresponding 95% confidence interval (CI) lay within the prespecified tolerance interval of ± δ around the optimum of no difference (difference = 0). The analysis was carried out using the Schuirmann TOST (two one-sided t‑tests) equivalence test on the basis of a one-sample t‑test with α = 0.025 to one side. The non-inferiority margin was set at δ = 0.5 mm which means that an undercorrection of more than 0.5 mm would be classified as non-equivalent with the set-up. The direction of the corrections have been taken into account as the expansion of the maxillary arch and the compression of the mandibular arch use different non-inferiority margins (maxilla: 0.5 mm, mandible −0.5 mm). No α correction was performed. All statistical analyses were carried out using the statistical software SAS version 9.4 (SAS, Cary, NC, USA). The error of the method (EM) for the linear measurements was determined using Dahlberg’s formula with EM = 0.16 mm for repeated measurements [11].

## Results

**Fig. 2:**
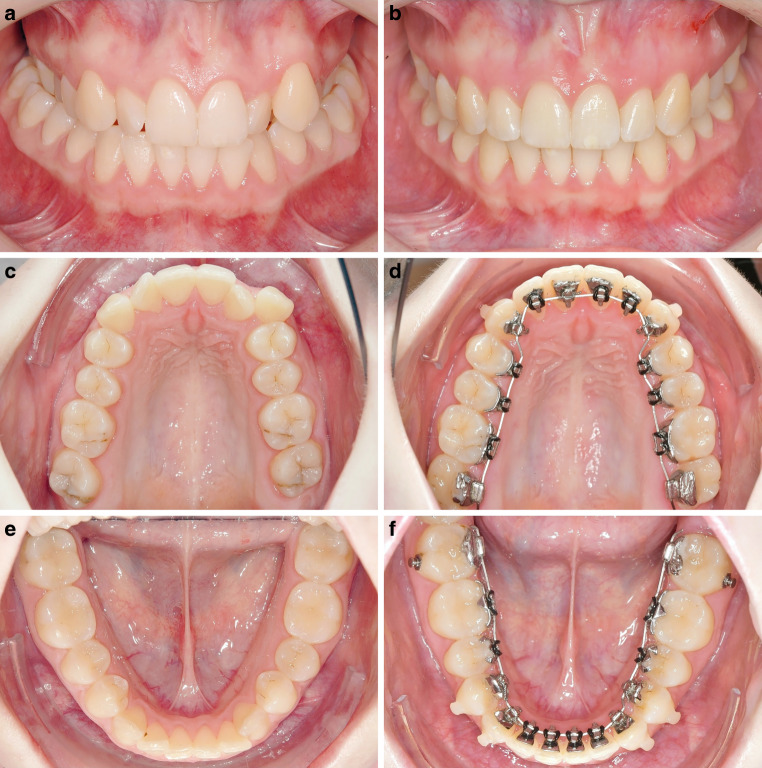
**a,** **c,** **e** Young patient with bilateral posterior crossbite at T1. Before lingual multibracket therapy the maxilla was expanded with rapid palatal expansion (RPE); **b,** **d,** **f** from T1 to T2 the maxillary arch was expanded 5.6 mm and the mandibular arch was compressed 4.3 mm (T2). The total treatment time with completely customized lingual appliances (CCLAs) was 1 year and 4 months **a,** **c,** **e** Junge Patientin mit beidseitigem Kreuzbiss zu Behandlungsbeginn (T1). Vor der Lingualbehandlung wurde der Oberkiefer mit einer GNE (Gaumennahterweiterung) expandiert. **b,** **d,** **f** Von T1 nach T2 wurden der Oberkiefer 5,6 mm expandiert und der Unterkiefer 4,3 mm komprimiert. Die Dauer der Behandlung mit der VILA (vollständig individuelle linguale Apparatur) betrug 1 Jahr und 4 Monate

**Fig. 3:**
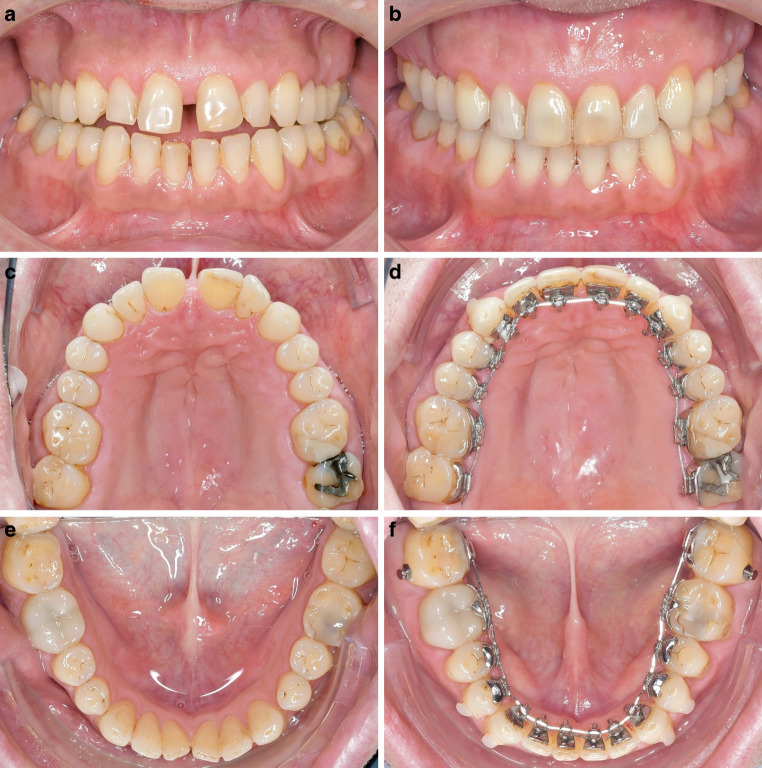
**a,** **c,** **e** A 49-year-old patient with bilateral posterior crossbite and frontal open bite at T1; **b,** **d,** **f** the total transverse correction achieved was 9 mm. The compression achieved in the mandibular arch was 7 mm. The total treatment time was 2 years and 5 months. Besides the crossbite, a half unit distal occlusion was corrected with class II elastics (see buccal button on 37 and 47) and the frontal open bite was closed after the transverse correction. The right frontal view picture shows the situation 2 years in the retention stage **a,** **c,** **e** 49-jähriger Patient mit beidseitigem Kreuzbiss und frontal offenem Biss zu Behandlungsbeginn (T1). **b,** **d,** **f** Es konnte eine transversale Korrektur von insgesamt 9 mm erzielt werden. Dabei betrug die Kompression im Unterkiefer 7 mm. Die festsitzende Behandlung dauerte 2 Jahre und 5 Monate. Neben dem seitlichen Kreuzbiss wurde der beidseitige Distalbiss mit Klasse-II-Gummizügen korrigiert (s. bukkales Knöpfchen an 37 und 47). Der frontal offene Biss konnte nach der Kreuzbissüberstellung geschlossen werden. Die rechte Abbildung der Frontalansicht zeigt die Situation 2 Jahre nach Abschluss der aktiven Behandlung

**Fig. 4:**
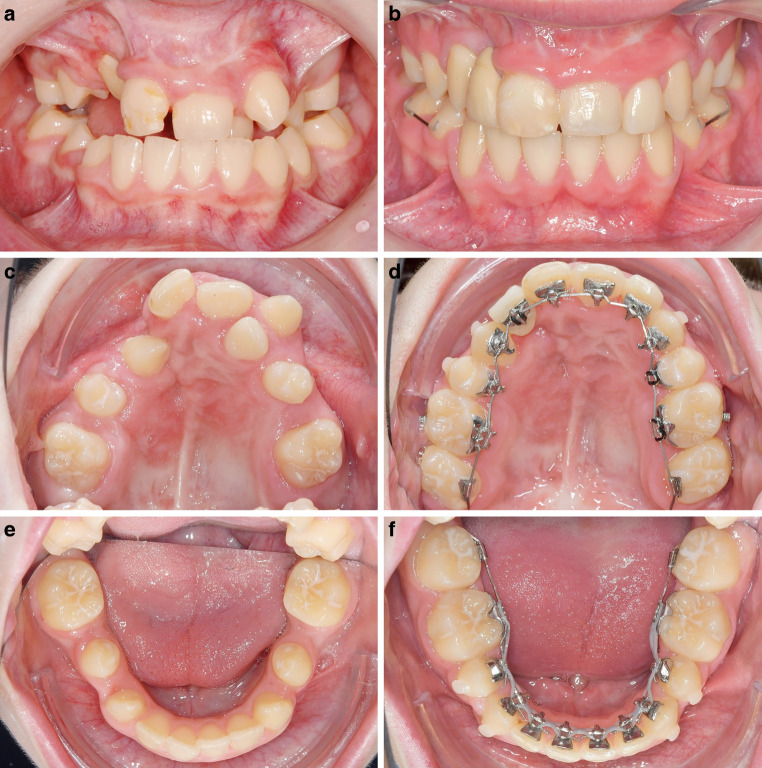
**a,** **c,** **e** Young patient with a cleft lip and palate. Teeth 12, 15, 25, 35 and 45 are missing; **b,** **d,** **f** after 2 years and 8 months of lingual treatment, the total transverse correction achieved was 9.7 mm. The compression in the mandibular arch was 3.5 mm. After orthodontic treatment, the missing 12 was replaced by a Maryland bridge bonded on 13 **a,** **c,** **e** Junger Patient mit Lippen-Kiefer-Gaumen-Spalte. Die Zähne 12, 15, 25, 35 und 45 sind nicht angelegt. **b,** **d,** **f** Während der 2 Jahre und 8 Monate dauernden Lingualbehandlung konnte eine transversale Korrektur von insgesamt 9,7 mm erzielt werden. Die Unterkieferkompression betrug 3,5 mm. Im Anschluss an die kieferorthopädische Behandlung wurde der fehlende Zahn 12 durch eine auf 13 angebrachte Maryland-Brücke ersetzt

After an average treatment time with lingual fixed appliances of 2.6 years (median 2.3, minimum/maximum 1.0/5.0, SD 0.98), in all 64 patients, the posterior crossbite was corrected successfully (Figs. 2, 3 and 4). Table 1 shows the corrections for both arches and the comparison with the set-up. The mean expansion achieved in the maxilla was 4.3 mm and the mean compression in the mandible 2.6 mm. The biggest maxillary expansion achieved was 11.3 mm and the biggest mandibular compression 7.3 mm. The mean total correction was 6.9 mm, with a maximum of 12.8 mm in one patient with a bilateral crossbite. Compared to the planned total transverse correction in the set-up, a mean overcorrection of 1.3 mm could be achieved. Table 2 shows the results of the equivalence test. With a non-inferiority margin of 0.5 mm the planned vs achieved corrections were equivalent in both jaws. Furthermore, the total correction achieved was equivalent to the total correction planned, with a final mean overcorrection that was more important in the mandible. Therefore, the null hypothesis was rejected.

**Table 1:** Descriptive analysis of the patient sample displaying age, achieved amount of transverse correction in both jaws and the comparison with the target set-up Deskriptive Analyse der untersuchten Patienten nach Alter, erzielter transversaler Korrektur in beiden Kiefern und Vergleich mit dem Ziel-Set-up

*N* = 64	Mean	SD	Median	Minimum	Maximum
Age at start (years)	23.5	13.70	17.0	9.0	63.0
T1 max. (mm)	40.4	5.85	41.6	26.2	54.4
T1 mand. (mm)	45.8	6.19	47.0	31.5	63.6
T2 max. (mm)	44.7	4.81	45.5	35.0	56.2
T2 mand. (mm)	43.2	5.54	44.3	31.5	57.8
Setup max. (mm)	44.4	4.46	45.2	35.0	53.5
Setup mand. (mm)	44.2	5.63	45.6	31.5	56.0
Expansion max. (mm)	4.3	2.55	3.70	−0.5	11.3
Compression mand. (mm)	2.6	2.15	2.35	−2.9	7.3
Total correction (mm)	6.9	2.41	6.7	3.3	12.8
Total correction setup (mm)	5.6	2.45	5.1	1.9	13.6
Overcorrection (mm)	1.3	1.24	1.4	−2.0	4.3

*SD* standard deviation, *max*. maxilla, *mand*. mandible

**Table 2:** Results of the Schuirmann TOST (two one-sided t‑tests) equivalence test on the basis of a one-sample t‑test with α = 0.025 to one side and a non-inferiority margin of δ = 0.5 mm Ergebnisse des TOST-Äquivalenztests nach Schuirmann auf der Basis eines einseitigen t‑Tests mit α = 0,025 und einer Nichtunterlegenheitsgrenze von δ = 0,5 mm

Variable	Mean (in mm)	95% lower CI	95% upper CI	Non-inferiority margin	*p*-value	Assessment: 95% CI ≤ margin
Maxilla: correction in setup-achieved correction	−0.3	–	*−0.05*	*0.5*	<0.0001	Equivalent
Mandible: correction in setup-achieved correction	1.0	*0.74*	–	**−***0.5*	<0.0001	Equivalent
Total correction (achieved total correction − setup total correction)	1.3	*0.97*	–	**−***0.5*	<0.0001	Equivalent

*95% CI* 95% confidence interval

**Fig. 5:**
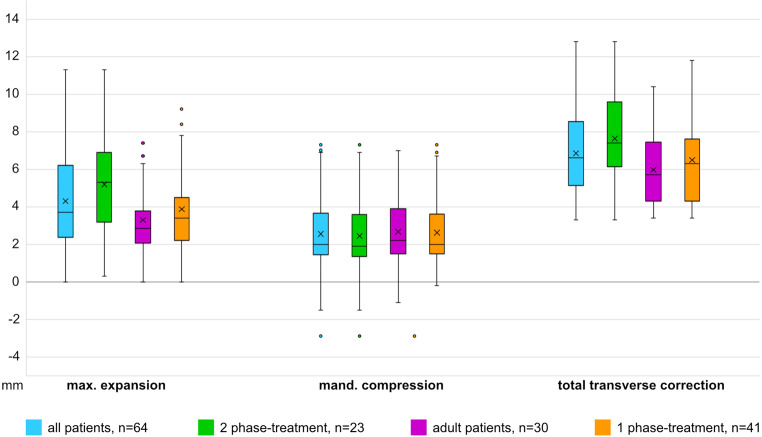
Transverse correction achieved in various age groups with or without pretreatment. *max.* maxilla, *mand.* mandible Erzielte transversale Korrektur in verschiedenen Altersstufen mit und ohne Vorbehandlung. *max*. Oberkiefer, *mand*. Unterkiefer

Figure 5 provides an overview of the treatment outcomes achieved across various age groups and their distribution. Of the 34 children and adolescents, 23 underwent two-phase treatment using an expansion appliance in the maxilla. The resulting mean expansion that was achieved is visibly bigger than in patients without that pretreatment. In adult patients, the transverse correction achieved in the maxilla and the mandible are of comparable extent.

## Discussion

In the past, scientists, mainly from Europe, pointed out that not only too narrow a maxilla, but also too broad a mandible has a causal part in a posterior crossbite [4, 21, 41]. This also holds for patients exhibiting cleft lip and palate or extreme orofacial dysfunction [12, 15, 18, 21, 43, 44]. A few recommendations aside, to the effect that this should be taken into account for malocclusion correction, clinical application of this scientific causality has remained neglected. Especially in a time when treatment with skeletally anchored appliances is highly popular (bone-borne SARPE, MARPE), the focus is on approaches that primarily look at the maxilla. This also means neglecting to an increasing extent the mandible, which almost always is too broad [1, 20, 39, 40]. A potential cause for this may possibly be a misinterpretation of results in studies on the stability of the mandibular archform in cases of anterior crowding. However, when a modification as small as possible of the intercanine distance is postulated for these cases, this relates exclusively to the anterior part of the dental arch [6, 19, 30].

In adult patients exhibiting posterior crossbite in particular, approaches that include surgical assistance (SARPE, MARPE) are frequently opted for, since the extent of the transverse correction that is required would exceed what is possible by dentoalveolar correction in the maxilla alone. This is also true for adults exhibiting marked distal occlusion along with a substantial transverse discrepancy. An additional intervention meant to surgically assist the transverse correction (SARPE) then precedes the surgical bite correction. On top of this added stress for the patient and the inherent general risk associated with it, SARPE treatment may lead to hardly predictable, in some cases severe complications, such as tooth darkening, periodontal recession, wound dehiscence, palatal fistula, and asymmetric expansion [3, 8, 10]. For the latter two in particular, a further surgical approach is then needed to address the problems [3, 10]. The results of this investigation demonstrate that with a crossbite correction approach involving both jaws even cases with more severe transverse discrepancies may be treated following the presented concept. The transverse correction that can be achieved is within the limits of what dentoalveolar compensation can perform. Even with a substantial transverse discrepancy of, for instance, 8 mm, the movement towards buccal or lingual in either jaw in the area of the crossbite would not exceed 2 mm per side. In the case of unilateral crossbite, a back-shifting of the mandible as a self-centring approach can assist in involving all four lateral areas of either arch in the transverse compensation [24]. In clinical practice, night-time wearing of unilateral cross elastics can assist with this shifting.

In children and adolescents, the expansion of the maxilla, in particular as rapid palatal expansion, preceding the multibracket treatment appears to make sense [9, 18]. In this study, too, the biggest total transverse correction was achieved in these patients. It has to be kept in mind, however, that in these cases, too, the crossbite correction as a whole should involve both jaws. Therefore, the crossbite may be under- rather than overcorrected at the beginning of the multibracket treatment. The maxillary expansion already achieved by the pretreatment has to be taken into account when designing the individual set-up. Furthermore, practitioners have to include the preliminary correction involving only the maxilla in their determination and selection of the relevant expansion and compression archwires.

**Fig. 6:**
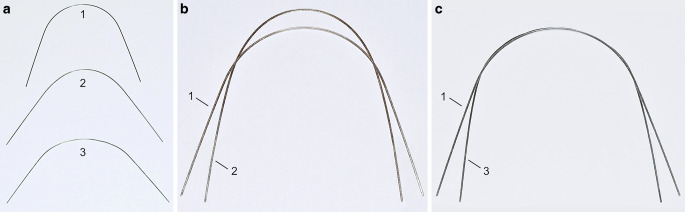
**a** Ideal labial archwire (*1*) and two wires with expansion bends in different locations. Expansion archwire with bends placed distal of the canine region (*2*), expansion archwire with bends placed in the anterior region from 3–3 (*3*); **b** If the expansion bends are placed distal of the canine (*2*) the wire will protrude the anterior teeth when inserted into a narrow maxillary arch; **c** If the expansion bends are placed in the anterior region from 3–3 (*3*), the anterior wire shape is not different from the ideal archwire shape when inserted into a narrow maxillary arch **a** Vestibulärer Idealbogen (*1*) und 2 vestibuläre Bögen mit Expansionsbiegungen, die in unterschiedlichen Bogenanteilen eingebogen wurden. Bei dem einen Expansionsbogen wurden die Biegungen distal der Eckzahnregion eingebogen (*2*); beim anderen Expansionsbogen wurden die Biegungen im anterioren Bereich zwischen den beiden Eckzähnen eingebogen. **b** Wenn die Expansionsbiegungen distal der Eckzähne eingebogen werden, wird der Bogen nach dem Einsetzen in den zu schmalen Oberkiefer die Frontzähne proklinieren. **c** Werden die Expansionsbiegungen im anterioren Bereich zwischen den beiden Eckzähnen eingebogen, erscheint der anteriore Bogenanteil nach dem Einsetzen in den zu schmalen Oberkiefer im Vergleich zum Idealbogen nicht verformt

The applied strategy underlying the design of the expansion and compression archwires has a decisive part in a successful treatment outcome. Simply selecting a broader or a narrower arch, as postulated in some older papers, has not proved very effective for posterior crossbite correction and, to achieve a noteworthy effect, has to be combined in clinical practice with the use of cross elastics [22, 31, 40]. As with all approaches that rely on compliance, the treatment outcome is then exclusively in the patient’s hands. To avoid this, the design of the archwires in question should not be based on the selection of a broader or narrower target shape of the archwire; rather, the ideal archwire shape expressed in the individual set-up should exhibit an overcorrection of the expansion or the compression only in the posterior segment. The correct method of incorporating the necessary bends for this correction is then of utmost importance. The bends should be incorporated exclusively in the anterior area, between the canines, and distributed as evenly as possible across the five interbracket distances (Fig. 1). As depicted in Fig. 6, incorporating the bends for correction distally from the canines would result, when the archwire is placed, in the immediate proclination of the anterior maxillary, and reclination of the anterior mandibular teeth with a marked overjet. This, of course, must be avoided.

The stability of the achieved transverse correction surely depends on various and numerous factors. After the correction of a lateral crossbite in particular, optimum interdigitation in the area of the posterior teeth is of particular importance and this can be achieved using CCLAs [16]. Furthermore, optimum interdigitation can be achieved much more easily when the compensation involves both jaws. With a movement towards buccal in the maxilla only, the result would be primary contact with the now low-protruding palatal cusps of the maxillary posterior teeth. Involving also the mandible in the correction can easily compensate for this disadvantage [1]. The very extent of the correction per jaw also has a part in the long-term stability, the probability of relapse increasing as the extent of tooth movement increases. In this respect, too, planning a correction that involves both jaws makes sense [4, 20, 41].

The decision as to what the right retention devices are should also include considerations on tongue position and function. A combination of fixed retainers in the anterior area with removable appliances meant to retain the outcome of transverse correction in the posterior area has proved favourable. Simple thermoformed trays are mainly not sufficiently stable, even when designed with a palatal coverage. Therefore, it is preferable, also in the mandible to use more rigid devices. The retaining elements of a simple mandibular plate will block the posterior teeth from movements towards labial. Slight modifications of an ordinary retention plate ideally have the potential to influence the position and function of the tongue [7, 13, 38]. The mandibular plate then acts at the same time as a “tongue lifter”. To assist the tongue further in adapting, it makes sense to select a removable retainer for the maxilla which does not block the tongue’s immediate contact with the palatal mucosa [7, 14, 18, 20, 37]. In any case, the harmony of tongue function and tongue position that is an objective will be possible only if unobstructed nasal breathing can be achieved [23, 45]. Furthermore, myofunctional therapy can assist with the harmonization of orofacial functions [14, 20, 23, 35, 42, 45, 46].

## Limitations

The retrospective nature of this study is the result of its innovative approach to posterior crossbite correction [4, 21, 41]. In the process, a narrow interpretation of exclusion criteria (only 3 patients were excluded due to the surgical correction of a class III malocclusion that facilitated posterior crossbite correction) enabled an unbiased view of clinical reality.

While representing various age groups, included patients, as numbers of patients in the various subgroups are relatively high, are sufficient to allow that descriptive differentiation by age and pretreatment which makes it possible to identify trends.

While possible periodontal changes were not part of the evaluation, clear gingival recession in the area of the posterior teeth was not found in any of the cases. When the tooth movements required for transverse correction are distributed across all four involved quadrants, the movements in every single quadrant are small and rarely exceed 2 mm, even with marked transverse discrepancy pretreatment.

The kind of tooth movement (uncontrolled tipping, controlled tipping, translation) in the course of posterior crossbite correction was not evaluated in the present study either. Still, looking at the achieved results, one does not have the impression that a massive dentoalveolar compensation with extreme buccal tipping of the upper, and lingual tipping of the lower, molars occurred (Figs. 2, 3 and 4).

The long-term stability of the achieved outcomes was not assessed in this investigation. And yet, the overall approach of the treatment method combined with a retention stage optimized for functionality warrants favourable prognostics, at least prima facie [20, 23]. Nonetheless, the reader should keep in mind that this study presents only preliminary results.

## Conclusions

Correction of a posterior crossbite involving both arches, with maxillary expansion and mandibular compression, makes sense. CAD/CAM expansion and compression archwires are an efficient tool to achieve the desired correction in the posterior segment even in more severe cases.
